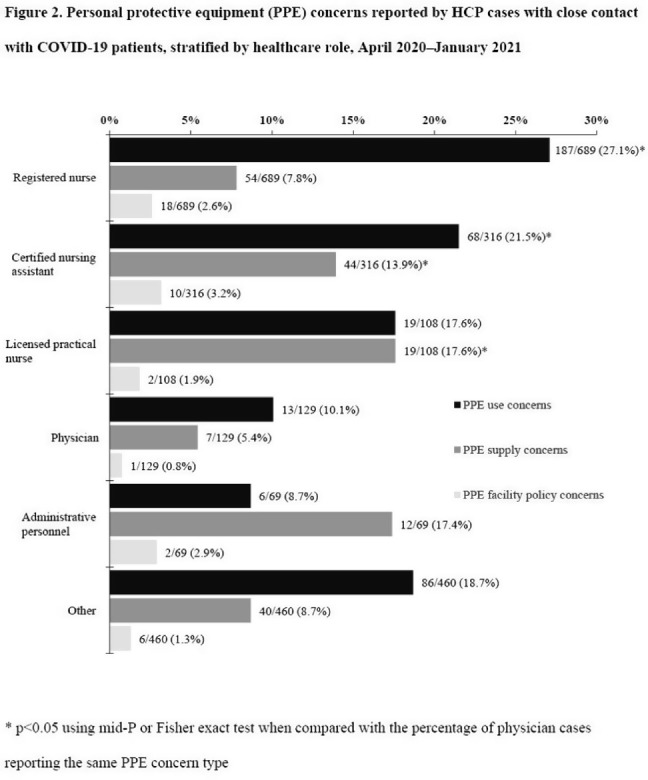# Characteristics of healthcare personnel who reported concerns related to PPE use during care of COVID-19 patients

**DOI:** 10.1017/ash.2022.68

**Published:** 2022-05-16

**Authors:** Nora Chea, Stephanie Tavitian, Cedric Brown, Taniece Eure, Rebecca Alkis, Gregory Blazek, Austin Penna, Joelle Nadle, Linda Frank, Christopher Czaja, Helen Johnston, Devra Barter, Kathleen Angell, Kristen Marshall, James Meek, Monica Brackney, Stacy Carswell, Stepy Thomas, Scott Fridkin, Lucy Wilson, Ashley Fell, Sara Lovett, Sarah Lim, Ruth Lynfield, Ruth SarahShrum, Erin C. Phipps, Marla Sievers, Ghinwa Dumyati, Cate Concannon, Kathryn McCullough, Woods, Sandhya Seshadri, Christopher Myers, Rebecca Pierce, Valerie Ocampo, Judith Guzman-Cottrill, Gabriela Escutia, Monika Samper, Sandra Pena, Cullen Adre, Tiffanie Markus, Kathryn Billings, Matthew Groenewold, Ronda Sinkowitz-Cochran, Shelley Magill, Cheri Grigg, Betsy Miller

## Abstract

**Background:** Healthcare facilities have experienced many challenges during the COVID-19 pandemic, including limited personal protective equipment (PPE) supplies. Healthcare personnel (HCP) rely on PPE, vaccines, and other infection control measures to prevent SARS-CoV-2 infections. We describe PPE concerns reported by HCP who had close contact with COVID-19 patients in the workplace and tested positive for SARS-CoV-2. **Method**: The CDC collaborated with Emerging Infections Program (EIP) sites in 10 states to conduct surveillance for SARS-CoV-2 infections in HCP. EIP staff interviewed HCP with positive SARS-CoV-2 viral tests (ie, cases) to collect data on demographics, healthcare roles, exposures, PPE use, and concerns about their PPE use during COVID-19 patient care in the 14 days before the HCP’s SARS-CoV-2 positive test. PPE concerns were qualitatively coded as being related to supply (eg, low quality, shortages); use (eg, extended use, reuse, lack of fit test); or facility policy (eg, lack of guidance). We calculated and compared the percentages of cases reporting each concern type during the initial phase of the pandemic (April–May 2020), during the first US peak of daily COVID-19 cases (June–August 2020), and during the second US peak (September 2020–January 2021). We compared percentages using mid-*P* or Fisher exact tests (α = 0.05). **Results:** Among 1,998 HCP cases occurring during April 2020–January 2021 who had close contact with COVID-19 patients, 613 (30.7%) reported ≥1 PPE concern (Table 1). The percentage of cases reporting supply or use concerns was higher during the first peak period than the second peak period (supply concerns: 12.5% vs 7.5%; use concerns: 25.5% vs 18.2%; p **Conclusions:** Although lower percentages of HCP cases overall reported PPE concerns after the first US peak, our results highlight the importance of developing capacity to produce and distribute PPE during times of increased demand. The difference we observed among selected groups of cases may indicate that PPE access and use were more challenging for some, such as nonphysicians and nursing home HCP. These findings underscore the need to ensure that PPE is accessible and used correctly by HCP for whom use is recommended.

**Funding:** None

**Disclosures:** None

**Table:**
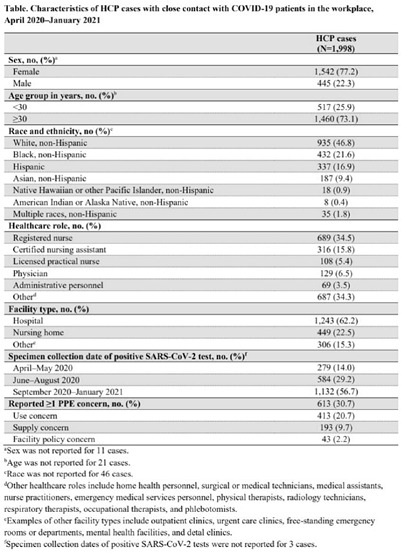

**Figure:**
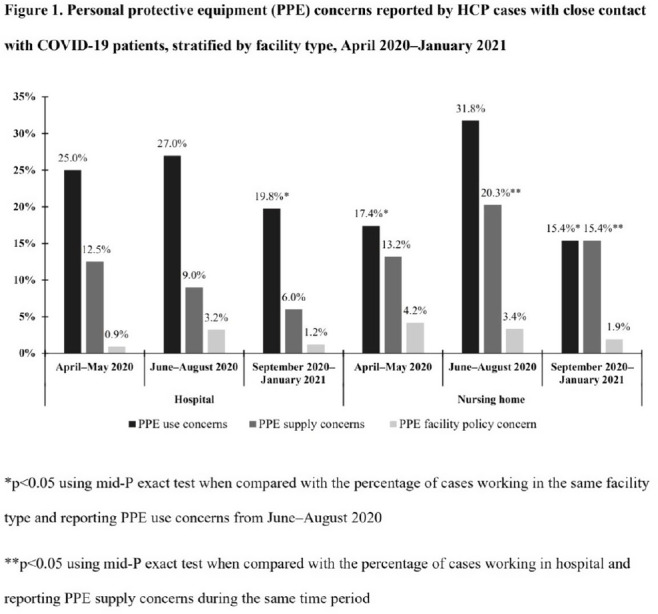

**Figure:**